# Rapid detection of the aspergillosis biomarker triacetylfusarinine C using interference-enhanced Raman spectroscopy

**DOI:** 10.1007/s00216-020-02571-2

**Published:** 2020-03-14

**Authors:** Susanne Pahlow, Thomas Orasch, Olga Žukovskaja, Thomas Bocklitz, Hubertus Haas, Karina Weber

**Affiliations:** 1 Institute of Physical Chemistry and Abbe Center of Photonics, Friedrich Schiller University Jena, Helmholtzweg 4, 07743 Jena, Germany; 2 InfectoGnostics Research Campus Jena, Centre for Applied Research, Philosophenweg 7, 07743 Jena, Germany; 3 Leibniz Institute of Photonic Technology - Member of the research alliance “Leibniz Health Technologies”, Albert-Einstein-Straße 9, 07745 Jena, Germany; 4 Department of Molecular and Applied Microbiology, Leibniz Institute for Natural Product Research and Infection Biology (HKI), Beutenbergstraße 11a, 07745 Jena, Germany; 5 Institute of Molecular Biology/Biocenter, Medical University of Innsbruck, Innrain 80-82, 6020 Innsbruck, Austria

**Keywords:** Raman spectroscopy, Interference-enhanced Raman spectroscopy, Siderophores, Aspergillosis, Infectious diseases, Biomarkers

## Abstract

Triacetylfusarinine C (TAFC) is a siderophore produced by certain fungal species and might serve as a highly useful biomarker for the fast diagnosis of invasive aspergillosis. Due to its renal elimination, the biomarker is found in urine samples of patients suffering from *Aspergillus* infections. Accordingly, non-invasive diagnosis from this easily obtainable body fluid is possible. Within our contribution, we demonstrate how Raman microspectroscopy enables a sensitive and specific detection of TAFC. We characterized the TAFC iron complex and its iron-free form using conventional and interference-enhanced Raman spectroscopy (IERS) and compared the spectra with the related compound ferrioxamine B, which is produced by bacterial species. Even though IERS only offers a moderate enhancement of the Raman signal, the employment of respective substrates allowed lowering the detection limit to reach the clinically relevant range. The achieved limit of detection using IERS was 0.5 ng of TAFC, which is already well within the clinically relevant range. By using an extraction protocol, we were able to detect 1.4 μg/mL TAFC via IERS from urine within less than 3 h including sample preparation and data analysis. We could further show that TAFC and ferrioxamine B can be clearly distinguished by means of their Raman spectra even in very low concentrations.

**Electronic supplementary material:**

The online version of this article (10.1007/s00216-020-02571-2) contains supplementary material, which is available to authorized users.

## Introduction

Invasive aspergillosis (IA) is one of the most common invasive fungal infections, mainly affecting immunocompromised patients and predominantly caused by the mold *Aspergillus fumigatus* [1]. This severe infection is associated with 90-day mortality rates of more than 60% depending on the patient cohort [2]. One reason for these high mortality rates is the challenging diagnosis of *Aspergillus* infections. There is no single “gold standard” for IA diagnosis and the detection of biomarkers focuses mainly on *Aspergillus* cell wall components like galactomannan or 1,3-β-D-glucan. Therefore, antimold prophylaxis is now widely applied to patients at risk, which further decreases the sensitivity of the employed diagnostic tests [3, 4].

To overcome the iron limitation by the host during infection, *Aspergillus* species secrete siderophores to satisfy their need for this essential nutrient. Siderophores are low molecular mass ferric iron-specific chelators, which have been shown to be an essential virulence factor of *A. fumigatus* [5–7]. The major extracellular siderophore of this mold is triacetylfusarinine C (TAFC) [8]. The exchange of iron in the [Fe]TAFC complex with the radionuclide ^68^Gallium and intravenous injection of this compound in mice infected with *A. fumigatus* showed an accumulation of the ^68^Gallium-TAFC in the infected parts of the lung, suggesting this modified siderophore as a useful tool for in vivo molecular imaging of *Aspergillus* infections [9]. Furthermore, these studies also showed a rapid renal clearance of the injected siderophore, which allows the detection of TAFC in urine samples for diagnosis of IA. Previously, TAFC was already detected in the blood of mice and the urine of rats infected with *A. fumigatus* [10, 11] and also in urine and bronchoalveolar lavage fluid samples of IA patients [12, 13], employing mass spectrometric methods for the detection in each of these studies.

Within this study, we investigate the potential of Raman microspectroscopy for the rapid detection of TAFC from urine samples. Raman spectroscopy provides a very high specificity due to the chemical information concealed in the fingerprint region of the spectra which allows the unambiguous identification of various analytes and biomarkers [14]. Acquiring spectra is usually achieved within seconds and nowadays cost-efficient portable handheld devices are available, enabling the use of Raman spectroscopy for point-of-care applications [15–20]. However, due to the intrinsically weak Raman scattering process, it is associated with a comparably low sensitivity [21]. Depending on the sample composition, there are various solutions to overcome this seemingly important drawback. For example, by employing a combination of a Raman spectrometer and a microscope, the investigation of single bacterial cells is easily possible [22–24]. Sample preparation techniques which aim at enriching the target cells or analytes are frequently employed as well to improve the overall sensitivity of the Raman spectroscopic assay [25, 26]. Furthermore, enhanced varieties of Raman spectroscopy, such as surface-enhanced Raman spectroscopy (SERS) [27, 28] or resonance Raman spectroscopy (RRS) [29], provide sensitivities several orders of magnitude higher than conventional Raman spectroscopy. While RRS requires an excitation wavelength that coincides with an electronic transition in the analyte molecule, SERS techniques exploit metallic nanostructures for local field enhancement. Especially for biomarker detection, SERS shows great promise [30]. The biomarkers are either identified via their SERS fingerprint [28] or indirectly using so-called SERS tags [31], which consist of a specific recognition element for the analyte and a metallic nanoparticle decorated with a Raman dye. While SERS is clearly superior towards conventional Raman spectroscopy regarding sensitivity, the storage and reproducibly of the required SERS substrates or nanoparticles can be an obstacle in routine applications [32]. For this reason, we applied either conventional Raman microspectroscopy or interference-enhanced Raman spectroscopy (IERS) for establishing a Raman-based detection scheme for the biomarker TAFC. IERS makes use of the interference phenomenon, occurring when light is passing through a thin transparent layer on a reflecting surface. For certain relations between thickness *d*, light wavelength λ, and index of refraction of the layer *n* (e.g., d = λ/4*n*), the transmitted and the reflected part of the light interferes constructively and the electric field strength is almost doubled. Accordingly, IERS only offers a moderately enhanced Raman signal due to an interference mechanism [33], but the substrates are easier to fabricate and long-term storage under ambient conditions is possible. In addition to that, the enhancement is distributed evenly throughout the entire substrate, which is beneficial in terms of the reproducibility of the results. The goal of this work is the Raman spectroscopic characterization of TAFC and to establish a Raman-compatible isolation protocol enabling the detection of this IA biomarker from urine samples in less than 3 h. To the best of our knowledge, there is no previous study addressing the Raman spectroscopic detection of TAFC.

## Materials and methods

### Siderophores

10^6^ spores/mL of *A. fumigatus* were grown for 36 h at 37 °C in *Aspergillus* minimal medium [34] shaking cultures containing 20 mM glutamine and 1% *w*/*v* glucose as sole nitrogen and carbon source, respectively. Supplementation of iron was omitted to achieve iron starvation conditions and thereby induce siderophore production. TAFC was subsequently purified from these culture supernatants by reversed-phase HPLC as described previously [35]. For isolation of [Fe]TAFC, the culture supernatants were saturated with FeCl_3_ to a final concentration of 1 mM before HPLC purification. Desferrioxamine B was purchased from Sigma-Aldrich (D9533) as mesylate salt.

### Raman spectroscopic measurements

The Raman measurements were performed in backscattering geometry using a Raman microscope (WITec GmbH, Ulm, Germany) with 514 nm excitation wavelength using a × 20 magnification objective (Zeiss Plan-Neofluar, × 20, NA = 0.4, Oberkochen, Germany). The laser spot size is approximately 1.6 μm. Detailed information on the setup can be found elsewhere [36]. The integration time per spectrum was 1 s, while the laser power was 10 mW. For the concentration series for each concentration, three scans (70 μm × 70 μm, 20 × 20 spectra) in one dried droplet were performed. The dried TAFC droplets will exhibit a coffee ring effect. We performed the scans within the spot ca. 10 μm away from the outer margin. In the classification data set with [Fe]TAFC and FerB, we included four samples per analyte with different concentrations. From each sample, four dried droplets were investigated, two on IER substrates and two on standard Raman chips. Per droplet, three line scans (ca. 80 μm) with 50 spectra were performed. As standard Raman chips, silicon wafers sputtered with aluminum were used [37]. For the IERS measurements, an additional layer of 60 nm Al_2_O_3_ was deposited on the aluminum surface. Further details can be found elsewhere [38].

### Isolation of TAFC from urine samples

Urine from healthy volunteers was sterilized via filtration using a 0.2-μm syringe filter. Three hundred microliters of the sterilized urine sample was transferred into a 1.5-mL reaction vial and 10 or 50 μL of 50 μg/mL [Fe]TAFC stock solution was added. In order to make sure no desferri-TAFC is present in the sample, 10 μL of 10 mM FeSO_4_ was pipetted to the mixture. With purified water, the volume of each sample was adjusted to 360 μL. Accordingly, concentrations of approximately 1.4 μg/mL or 7.1 μg/mL are obtained. For the negative control, purified water was used instead of [Fe]TAFC stock solution. For extraction of TAFC, first 300 μL of a 1:1 mixture of chloroform (CHCl_3_ stabilized with 0.6% ethanol) and diethyl ether were added to the urine sample and mixed thoroughly. Subsequently, the sample was centrifuged at 20.000 rcf at 4 °C for 3 min and the organic phase was removed. Then, the aqueous phase was mixed with 300 μL CHCl_3_ and another centrifugation step (parameters as previously described) was performed. The organic phase was collected in a new vial and the previous steps (adding 300 μL CHCl_3_, centrifugation, and collection of organic phase) were repeated twice. The combined organic phases were heated up to 60 °C until the solvent was completely evaporated. The residue was resolubilized in 10 μL purified water. 3 × 1 μL per sample was applied onto the Raman chips and dried before the Raman measurements were conducted. This step can be either carried out at room temperature or sped up by placing the substrate in an oven at 70 °C for 5 min. For the Raman analysis of the urine samples, three line scans (80 μm, 50 spectra) were acquired per sample.

### Data processing

The data processing was performed using an in-house developed script in the programming language R [39]. The Raman spectra were background corrected using the sensitive nonlinear iterative peak (SNIP) clipping algorithm using 40 iterations and second-order clipping filter [40]. Next, if it was required, the spectra were vector normalized. For spectral comparison across the groups, mean Raman spectra for each group were calculated using preprocessed spectra of all samples. Aiming to perform automatic differentiation between the TAFC and FerB siderophores, multivariative data analysis was performed. To that end, first, for all spectra, wavenumber axis calibration was performed using a Raman spectrum of 4-acetamidophenol as a reference, which was collected on each day of measurements. Next, the Raman spectra were background corrected, vector normalized, and used as input for a principal component analysis (PCA), which was performed to reduce the dimensionality of the data while retaining the most significant information for classification. The PCA was followed by a linear discriminant analysis (LDA), which was employed to build a classification model. Additionally, to evaluate stability and robustness of the model, different concentrations of the siderophores measured on different days were considered as (technical) replicates and a leave-one-concentration-out-cross-validation (LCOCV) was performed [41]. In this method, the spectra of one concentration were held out from the data set, and the LDA model was redeveloped using the remaining spectra. The resulting model was then used to predict the spectra of the removed concentration. This process was repeated with every concentration until all spectra were classified.

## Results and discussion

**Fig. 1:**
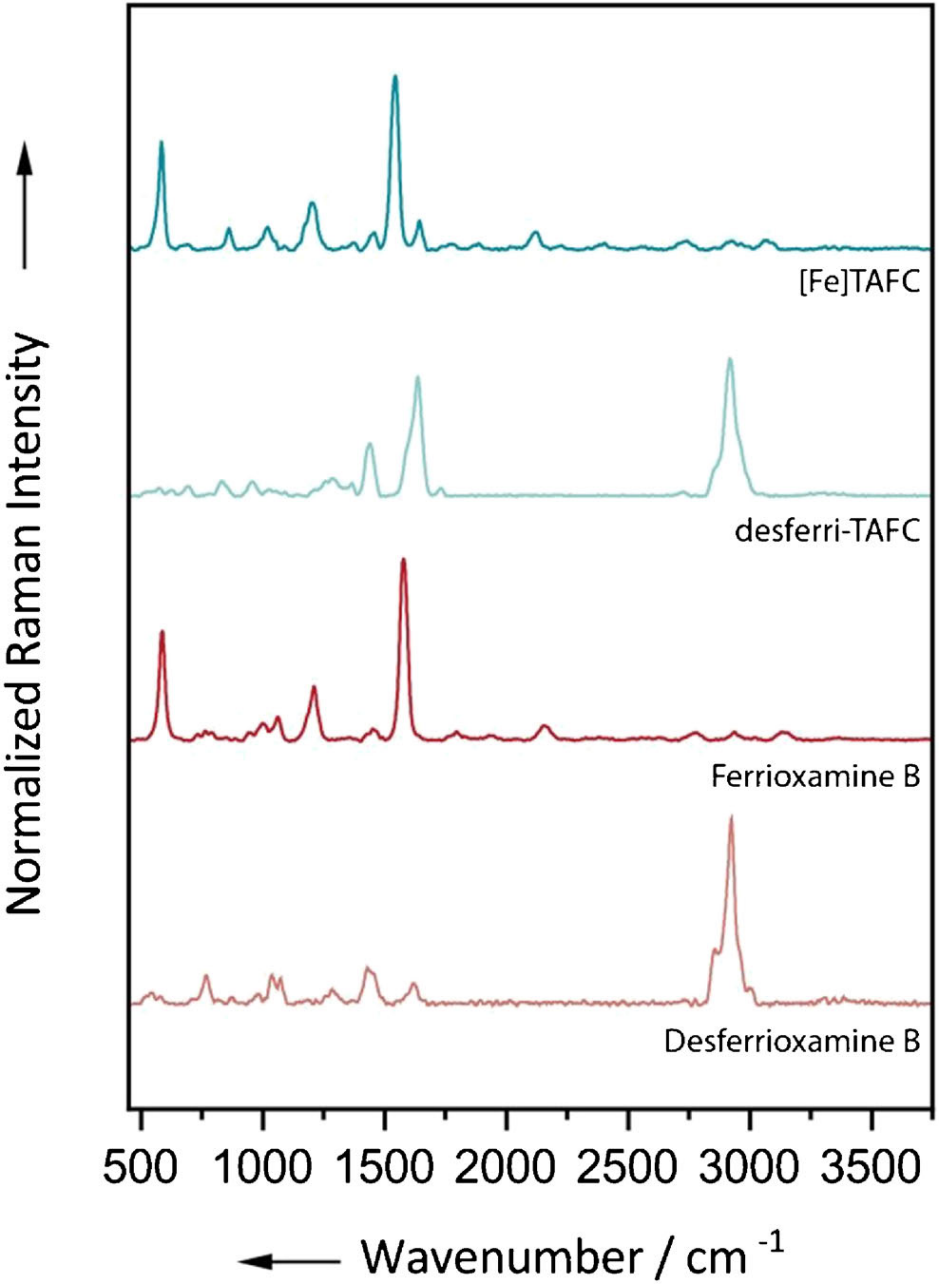
Normalized Raman spectra of desferri-TAFC and DesfB and their iron complexes. The bands in the C–H stretching region in the spectra of the iron complexes are hardly visible due to the resonance enhancement of the bands in the fingerprint region. The spectra were shifted vertically for clarity

As a first step towards establishing a protocol for the spectroscopic detection of the biomarker TAFC, reference spectra were recorded, since neither Raman spectra of the iron complex nor the corresponding desferri form exist in the literature to date. The spectra were acquired with an excitation wavelength of 514 nm from drop-dried 500 μM aqueous solutions of the corresponding compounds. Furthermore, Raman spectra of desferrioxamine B (DesfB) and its iron complex (ferrioxamine B (FerB)) were recorded, because this siderophore is structurally related to TAFC [42]. As can be seen in Fig. S1 of the Electronic Supplementary Material (ESM), the selected excitation wavelength coincides with the absorption bands of [Fe]TAFC and ferrioxamine B. Note that the UV-Vis spectra where acquired in aqueous solution, while the Raman measurements are conducted on dried residues. However, the dried compounds display approximately the same orange-brownish color as the corresponding solution. Accordingly, Raman bands associated with the chromophoric system of the molecules will be enhanced due to the resonance effect. The normalized Raman spectra are shown in Fig. 1. The Raman spectra of both desferri-TAFC and DesfB exhibit a wide CH band with a maximum at 2915 cm^−1^, or 2925 cm^−1^ respectively, which can be assigned to the asymmetric stretching vibrations of CH_2_ groups. Different shoulders of the band are due to further (symmetric and asymmetric) stretching vibrations of CH_2_ and CH_3_ groups. The very intense band at 1652 cm^−1^ in the desferri-TAFC spectrum probably results from the carbonyl groups of the hydroxamate, amide, and ester functionalities (amide I band) present in the molecule. In the DesfB spectrum, the amide I band appears at 1624 cm^−1^ [43]. Furthermore, the desferri-TAFC spectrum features a distinct band at 1441 cm^−1^ which is in a range characteristic for C–H deformation vibrations. The corresponding peak in the DesfB spectrum can be found at 1447 cm^−1^. The less intense bands at approximately 1300 cm^−1^ can be assigned to C–N valence vibrations (amide II band) of amide groups in the desferri-TAFC and DesfB molecules. The superimposed peaks at 1048 and 1088 cm^−1^ probably originate from stretching vibrations of C–C and N–O units in DesfB. In contrast to the desferri variants, the Raman spectra of the TAFC iron complex and ferrioxamine (FerB) do not have a pronounced CH stretching range. This is due to the coloration of the iron form of the two compounds, which results in a resonance enhancement of many bands in the fingerprint region. While the C–H bonds and their corresponding vibrations are not affected by the formation of the iron complexes, the oxygen atoms of the hydroxamate groups are directly coordinating the iron in the center of the metal complex. Therefore, the respective vibrations are enhanced, while the other bands appear decreased in comparison. The carbonyl valence vibration can be observed at 1647 cm^−1^ with medium intensity only in the TAFC spectrum, indicating an increased C=N bond character of the hydroxamate groups due to the iron complexation [44]. The C–N stretching vibration of the resonative O–C–N bonds appears very intense around 1550 cm^−1^ for both iron complexes. As expected, the Fe–O band at approximately 585 cm^−1^ can be observed at high intensity for the TAFC iron complex and FerB due to the resonance enhancement. More detailed spectra of the previously discussed compounds with marked peaks and the structural formulas are displayed in Figs. S2 and S3 of the ESM.

**Fig. 2:**
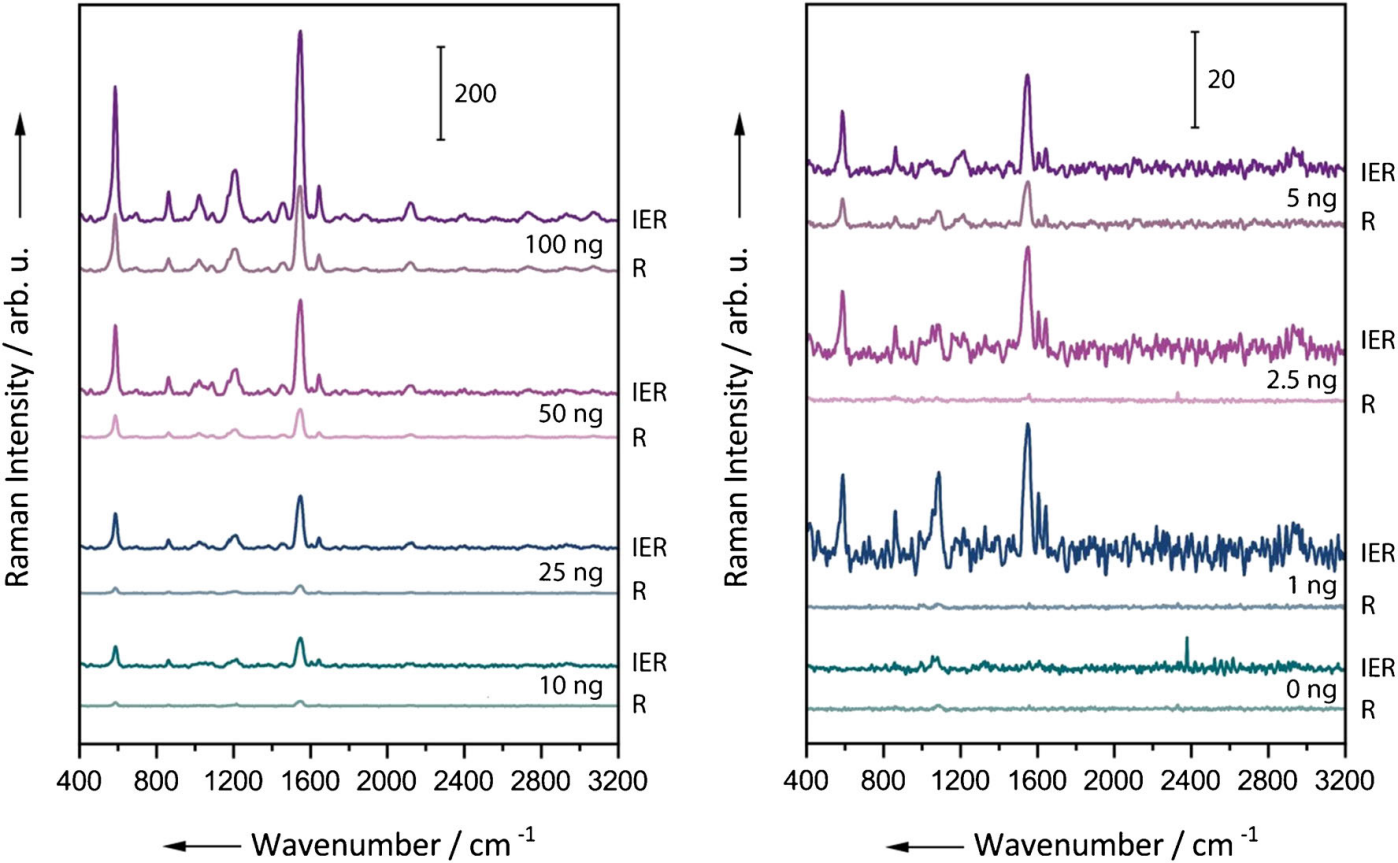
Raman (R) and interference-enhanced Raman (IER) mean spectra of drop-dried [Fe]TAFC samples. The amounts of [Fe]TAFC refer to a sample volume of 0.75 μL. A comparison of IER and Raman spectra shows that IER enables a more sensitive detection of smaller quantities. The spectra were shifted vertically for clarity

In order to compare the sensitivities for the detection of TAFC via conventional and interference-enhanced Raman spectroscopy, samples in a concentration range between approximately 1.5 and 150 μM were examined. Since for each measurement 0.75 μL sample volume was applied and dried on the Raman substrates, the exact concentrations were adjusted so that they correspond to total amounts of 1–100 ng [Fe]TAFC (see ESM Table S1). The aqueous [Fe]TAFC solutions were applied onto two different types of Raman substrates. A Raman chip with measuring fields made of aluminum was used for conventional Raman spectroscopy. The same chip variant with an additional 60 nm Al_2_O_3_ coating was employed for IERS. This specific thickness of the Al_2_O_3_ layer was optimized for an excitation wavelength of 514 nm and will generate an amplification of the Raman signal via constructive inference, thus enabling a more sensitive detection of the analyte [38]. Even though the enhancement depends on the wavenumber, the variation is small enough to provide sufficient enhancement over the entire investigated wavenumber region. (In [38], the wavenumber dependency of the enhancement factor is displayed.) In Fig. 2, the respective spectra of the dilution series are shown. Despite working with dried samples, a concentration dependency can be observed. A more detailed analysis can be found in ESM Fig. S4, where we plotted the peak areas for the Fe–O band at 583 cm^−1^ and the peak at 1550 cm^−1^ against the TAFC amount in the droplets. Comparing the *R*^2^ values of the different plots, it can be concluded that conventional Raman spectroscopy (*R*^2^ = 0.842 for 583 cm^−1^ and 0.696 for 1550 cm^−1^) can offer a slightly better linearity than IER (*R*^2^ = 0.658 for 583 cm^−1^ and 0.657 for 1550 cm^−1^). However, it should be noted that neither method provides a good linearity, which is most likely due to the measurement of drop dried samples. With conventional Raman spectroscopy, amounts down to 5 ng can be detected from the spectra with the naked eye. Due to the constructive interference effect, the IER substrates provide a higher sensitivity; with them, even the lowest investigated quantity of 1 ng can be identified as [Fe]TAFC via the marker bands at 583 cm^−1^ and 1550 cm^−1^ by the naked eye. The spectra in the lower concentration range clearly show the greater sensitivity of IER, but also a rather high level of noise. Unfortunately, not only the analyte signal is enhanced but also the background noise. Also, the fluorescent background increases when using the IER substrates.

As the expected amounts of extracted TAFC in the clinical samples can be as low as a few nanograms, we conducted further measurements with the IER substrates to optimize the measurement conditions for concentrations lower than 5 ng. We found that it is possible to detect down to 0.5 ng using interference-enhanced Raman scattering. The IER spectra for the lower concentrations can be found in the ESM (Fig. S5). We also calculated the limit of detection (LOD) based on the IUPAC definition that the LOD is equal to the signal of the blank sample plus three times of its standard deviation. The corresponding peak area analysis of ESM Fig. S4 showed that the LOD for IER is 0.4 ng (both for 583 cm^−1^ and 1550 cm^−1^ peak), which is slightly better than the one for the conventional Raman approach (2.4 ng for 583 cm^−1^ and 2.8 ng for 1550 cm^−1^).

Reported concentrations of [Fe]TAFC in IA patient urine range from 0.15 to 147 ng/mL [13]. With a sample volume of 300 μL, this resembles total amounts of about 0.05–49 ng [Fe]TAFC per sample. Assuming an extraction yield of 95%, this corresponds to quantities of 0.05–47 ng. In order to transfer the extracted TAFC onto the Raman substrate, the extracted compound is dissolved in a volume of 10 μL. If 1 μL of the dissolved extract is applied, quantities between 0.005 and 4.7 ng must be detectable. As previously shown, quantities in the nanogram range can only be detected with IER (LOD = 0.4 ng), so that at least the upper part of the clinically relevant range can be covered with this approach. Accordingly, all Raman measurements of the urine extracts were performed using only the IER substrates.

The extraction of [Fe]TAFC from the urine samples was carried out according to a modified version of the protocol previously described by Hoenigl et al. [13] In Fig. S6 (see ESM), the corresponding work process is depicted. First, a preextraction step with a 1:1 mixture of chloroform and diethyl ether is performed. Then, the [Fe]TAFC is extracted using chloroform as solvent. Finally, the solvent is evaporated and the residue is resolubilized in water, which is applied onto the IER substrate. For the successful Raman spectroscopic detection of the extracted [Fe]TAFC, it is advisable to use chloroform without amylene as stabilizer. Otherwise, the amylene will be enriched during the evaporation of the solvent and show up in the Raman spectra. Even though the TAFC maker bands will still be visible, the detection of lower TAFC concentrations might be hampered by the much stronger signals of the stabilizer.

This sample preparation protocol enables the Raman spectroscopic investigation of TAFC with only minimal interference from residual urine components, which might be carried along in small amounts during the extraction procedure. Urine contains a mixture of various salts, urobilin (which is partly responsible for the yellow color of the urine), and some fluorescent components [45–48]. Therefore, a successful Raman spectroscopic detection of [Fe]TAFC using drop-dried samples of this complex body fluid is very difficult, as a strong fluorescent background and Raman bands of other components will complicate the detection of [Fe]TAFC. Due to the small concentration of [Fe]TAFC in the urine, a measurement in liquid phase is not a feasible alternative. For comparison, we included spectra of liquid samples (urine and purified water) in the ESM (Fig. S7) that contained the amounts of TAFC as the samples used for the extraction. Only in the 50 μg/mL [Fe]TAFC stock solution we were able to observe a weak marker band at 1550 cm^−1^. We would like to point out that in principle, Raman measurements in the liquid phase can be advantageous since they enable the investigation of defined concentrations which allows a more reliable quantification. For example, Rohleder et al. demonstrated how Raman spectroscopy can be employed for the quantitative analysis of several analytes in blood sera, while Parachalil et al. established an ultrafiltration-based sample preparation protocol for blood sera that allows for Raman-based drug monitoring of busulfan and methotrexate [49, 50]. Even though these results indicate that multivariate analysis of Raman spectra directly acquired from complex body fluids is possible under certain conditions, for the detection of [Fe]TAFC in urine, this was not the case. In order to detect amounts in the lower nanogram range, some enhancement of the Raman signal is necessary. To that end, we employed IER for investigating drop-dried extracts of [Fe]TAFC.

**Fig. 3:**
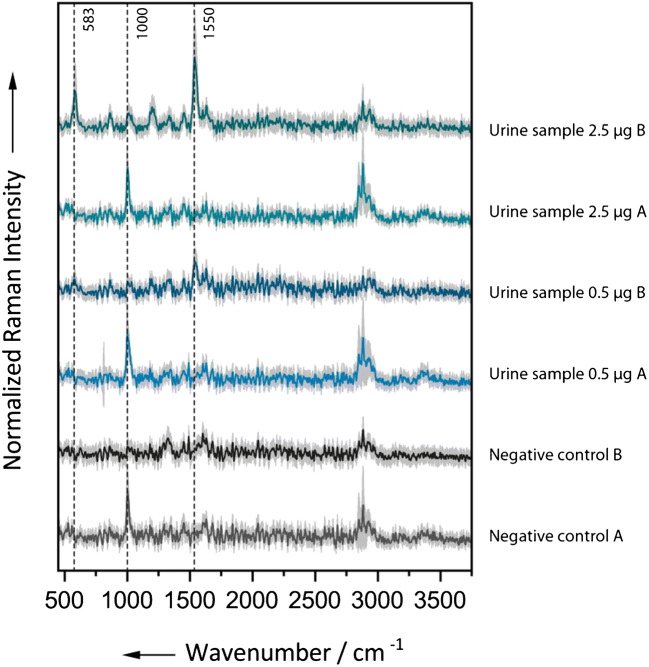
Background-corrected Raman spectra with standard deviation from the dried extracts obtained from urine samples with defined amounts of [Fe]TAFC. The spectra of residues from the preextraction step (A) with a chloroform/diethyl ether mixture feature a band at 1000 cm^−1^ indicating the presence of urea. Marker bands for [Fe]TAFC are only visible in the spectra from the chloroform extraction (B). Accordingly, it can be assumed, that the biomarker is mainly enriched in the pure chloroform phase. The spectra were shifted vertically for clarity

In order to test the applicability of the extraction protocol for a Raman-based detection scheme, we spiked urine samples from healthy volunteers with defined amounts of [Fe]TAFC. As negative control, a urine sample with no added [Fe]TAFC was included as well. After the previously described sample preparation had been performed, we recorded not only the spectra of the dried residues from the chloroform extract but also the spectra from those of the preextraction step. This was to verify that the analyte is not already partially enriched in the chloroform/diethyl ether phase (in the following referred to as extract A). The Raman spectra of the dried residues of extract A from all samples feature a more or less pronounced CH stretching band and a distinct band at approximately 1005 cm^−1^ indicating the presence of urea (see Fig. 3). The spectra acquired from extract B (the pure chloroform phase) showed peaks at 583 and 1550 cm^−1^ for the samples that had been mixed with 0.5 or 2.5 μg [Fe]TAFC respectively. The TAFC amounts correspond to concentrations of 1.4 μg/mL or 7.1 μg/mL. In the sample with 2.5 μg TAFC, more characteristic bands at 1025 and 860 cm^−1^ are clearly visible as can be seen in Fig. 3. In the negative control, we did not observe any peaks for [Fe]TAFC. Accordingly, the analyte can be identified unambiguously even after extraction from a complex matrix such as urine. However, the currently detectable amounts are still significantly higher than the concentrations typically found in clinical samples.

**Fig. 4:**
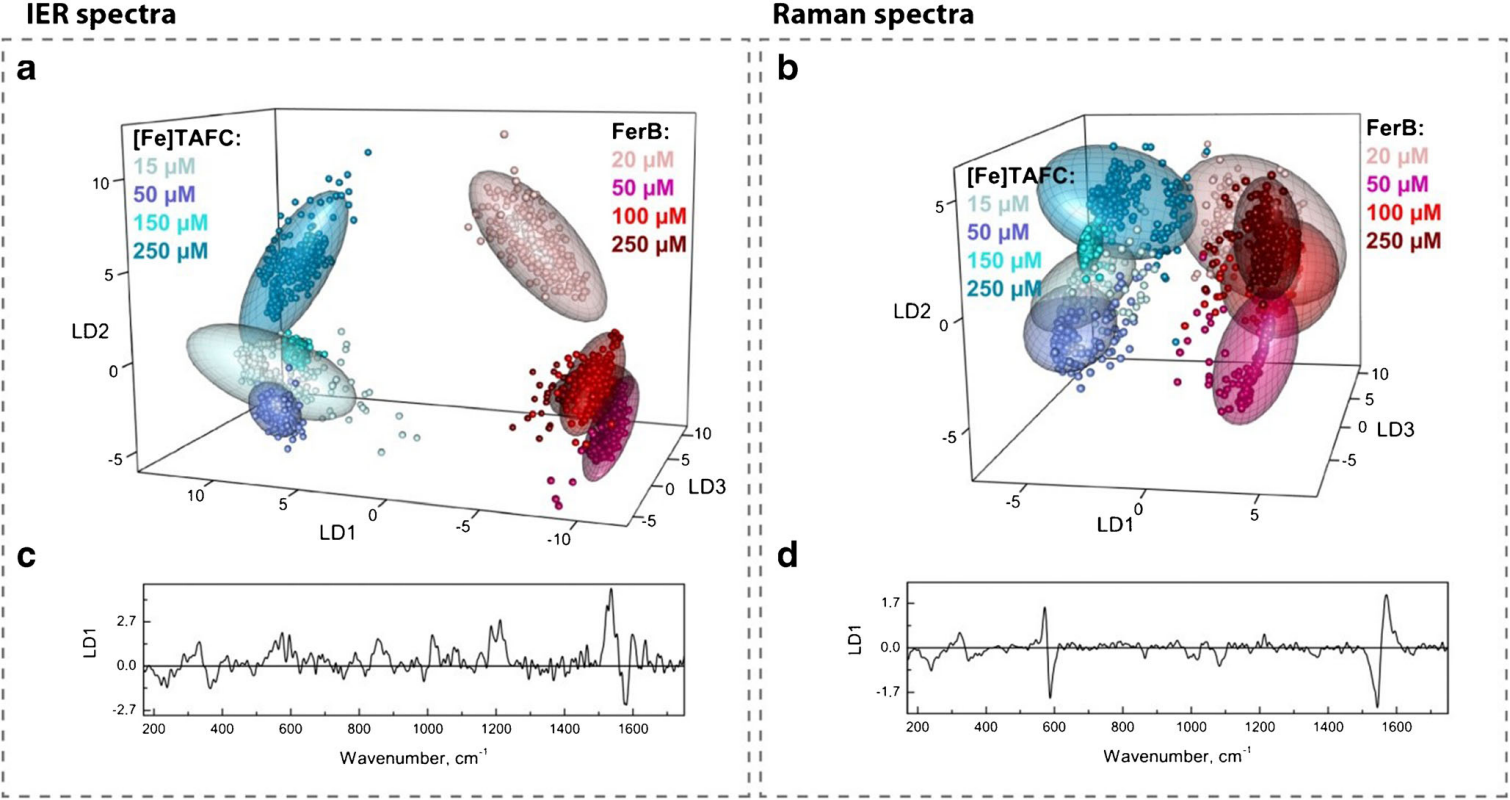
Linear discriminant analysis plot and first LDA loading vector showing the differentiation of [Fe]TAFC and FerB by means of their IER spectra (**a**, **c**) and Raman spectra (**b**, **d**). Independent from the concentration clear separation of two siderophores was achieved. Comparing both techniques, it can be concluded that IER provides a slightly better separation, since the spectra from the two different analytes are further apart in LDA space. Differentiation is mainly based on the shift of the Raman band around 1550 cm^−1^

Siderophores are excreted by various bacterial and fungal pathogens during infection and play an important role regarding virulence [51, 52]. Desferrioxamine B, for example, is a siderophore produced by *Streptomyces pilosus* [53]. Even though *Streptomyces* infections in humans are rare, it is used as medication due to its capability to chelate iron in case of acute iron poisoning or hemochromatosis [54, 55]. The iron-loaded desferrioxamine molecules are excreted via urine [56]. Therefore, it can occur that both siderophores are present in the patients’ urine, even if it is unlikely. Because of the structural similarities of [Fe]TAFC and ferrioxamine B, their Raman spectra can be hard to distinguish from one another by the naked eye at low concentrations. The predominant features for both analytes are the Fe–O peak around 580 cm^−1^ and the C–N stretching band around 1550 cm^−1^. Even though the C–N stretching band is shifted to higher wavenumbers for ferrioxamine B, we decided to conduct an exploratory data analysis to enable automated discrimination between samples with easy visual readout. Especially with regard to the Raman spectroscopic detection of TAFC as a diagnostic biomarker, a reliable identification is of great importance. For the classification model, we recorded Raman and IER spectra of [Fe]TAFC and ferrioxamine B in different concentrations (ESM Table S2) on different days. The corresponding Raman spectra are depicted in Fig. S8 (see ESM). The aim of the following PCA/LDA analysis mainly was to visualize how well IER and Raman perform in comparison regarding the discrimination of the two compounds. In order to build the respective classification model, at first, dimensionality of the data was reduced with PCA. Next, eight principal components were selected and used as input for the LDA. The resulting LDA plots are displayed in Fig. 4a, c. It can be recognized that independently from the concentration for both detection techniques, the Raman spectra of the two analytes are clearly separated. The differentiation can be explained by the first LDA loading vector, which is for IER plotted in Fig. 4b and for conventional Raman in Fig. 4d. We can see that, as expected, successful classification is based mainly on the shift of C–N stretching band around 1550 cm^−1^. Additionally, for the Raman data, the shift of the Fe–O peak around 580 cm^−1^ appears to be relevant as well. From the confusion tables, which are presented in Table S3 in the ESM, it can be seen that there is no misclassification between the IER spectra of [Fe]TAFC and FerB. Wrong predictions appear only for the different concentrations of the same analyte (ESM Table S3a). In the case of the Raman spectra, five spectra of FerB were misclassified as [Fe]TAFC, while five spectra of [Fe]TAFC were assigned to FerB group (ESM Table S3b). For the IER spectra, an overall accuracy (including the separation of the different concentrations) of 99% and for the Raman spectra of 97% was achieved. The slightly superior results for IER can be explained by the interference enhancement and the accordingly more pronounced spectral features. Additionally, to evaluate stability and robustness of the PCA-LDA model, a two-class classification with leave-one-concentration-out-cross-validation (LCOCV) was performed. For both techniques, already one principal component was enough to achieve perfect differentiation (100% accuracy both for Raman and IER) between [Fe]TAFC and FerB. Corresponding LDA plots are depicted in ESM Fig. S9 together with their loading vectors, which again confirm that the successful differentiation is mainly due to the shift of the Raman band at 1550 cm^−1^.

## Summary and conclusion

Overall, the results for Raman spectroscopic detection of TAFC are very promising in terms of a future application for the diagnosis of invasive aspergillosis. We demonstrated that interference-enhanced Raman spectroscopy is a valuable tool for the sensitive detection of biomolecules such as siderophores. Even though the enhancement factors are much lower compared with SERS, we could show that the sensitivity of the detection method is already sufficient to detect some clinically relevant amounts. Further benefits of IERS are the long-term stability and the uncomplicated fabrication of the substrates, as well as the uniform signal enhancement over large areas. The Raman spectroscopic detection of TAFC from urine samples down to a concentration of 1.4 μg/mL was enabled using an extraction protocol. Even from this complex sample matrix, typical Raman spectra of the biomarker were obtained within less than 3 h. By means of chemometric analysis, a reliable differentiation of TAFC and structurally related bacterial siderophore ferrioxamine B was achieved.

## Electronic supplementary material

ESM 1 (DOCX 1.87 mb)
