# Supplementary material for: Rapid detection of the aspergillosis biomarker triacetylfusarinine C using interference-enhanced Raman spectroscopy
Source: Anal Bioanal Chem. 2020 Mar 14;412(24):6351–60. doi: 10.1007/s00216-020-02571-2 (PMC7442771; doi:10.1007/s00216-020-02571-2)
Supplement: Supplementary file 1 — (DOCX 1.87 mb) [file 216_2020_2571_MOESM1_ESM.docx]

Analytical and Bioanalytical Chemistry

Electronic Supplementary Material

**Rapid detection of the aspergillosis biomarker triacetylfusarinine C using interference**-**enhanced Raman spectroscopy**

Susanne Pahlow, Thomas Orasch, Olga Žukovskaja, Thomas Bocklitz, Hubertus Haas,
Karina Weber

**Contents**

- Figure S1. UV-Vis spectra of [Fe]TAFC and ferrioxamine B measured in solution.
- Figure S2. Raman spectra and structural formulas of ferrioxamine B and desferrioxamine B.
- Figure S3. Raman spectra and structural formulas of [Fe]TAFC and desferri-TAFC.
- Table S1. [Fe]TAFC masses, amounts of substance and concentrations for investigated samples.
- Figure S4. The peak area of the 583 cm^-1^ and 1550 cm^-1^ Raman mode as a function of [Fe]TAFC amount in the dried droplet with linear fitting.
- Figure S5. Interference enhanced Raman (IER) mean spectra of drop dried of [Fe]TAFC samples.
- Figure S6. Schematic display of the extraction protocol for [Fe]TAFC from urine samples.
- Figure S7. Background corrected Raman mean spectra of [Fe]TAFC acquired in solution: a) urine, b) purified water
- Table S2. [Fe]TAFC and ferrioxamine B masses, amounts of substance and concentrations for classification analysis.
- Figure S8. Fingerprint regions of a) IER spectra and b) Raman spectra used for the differentiation of TAFC and FerB.
- Table S3. PCA/LDA classification results for a) IER spectra and b) Raman spectra.
- Figure S9. Mean spectra of [Fe]TAFC and FerB, LDA vector for two class classification with leave-one-concentration-out-cross-validation and corresponding LDA plot for IER spectra (a, b, c) and Raman spectra (d, e, f).


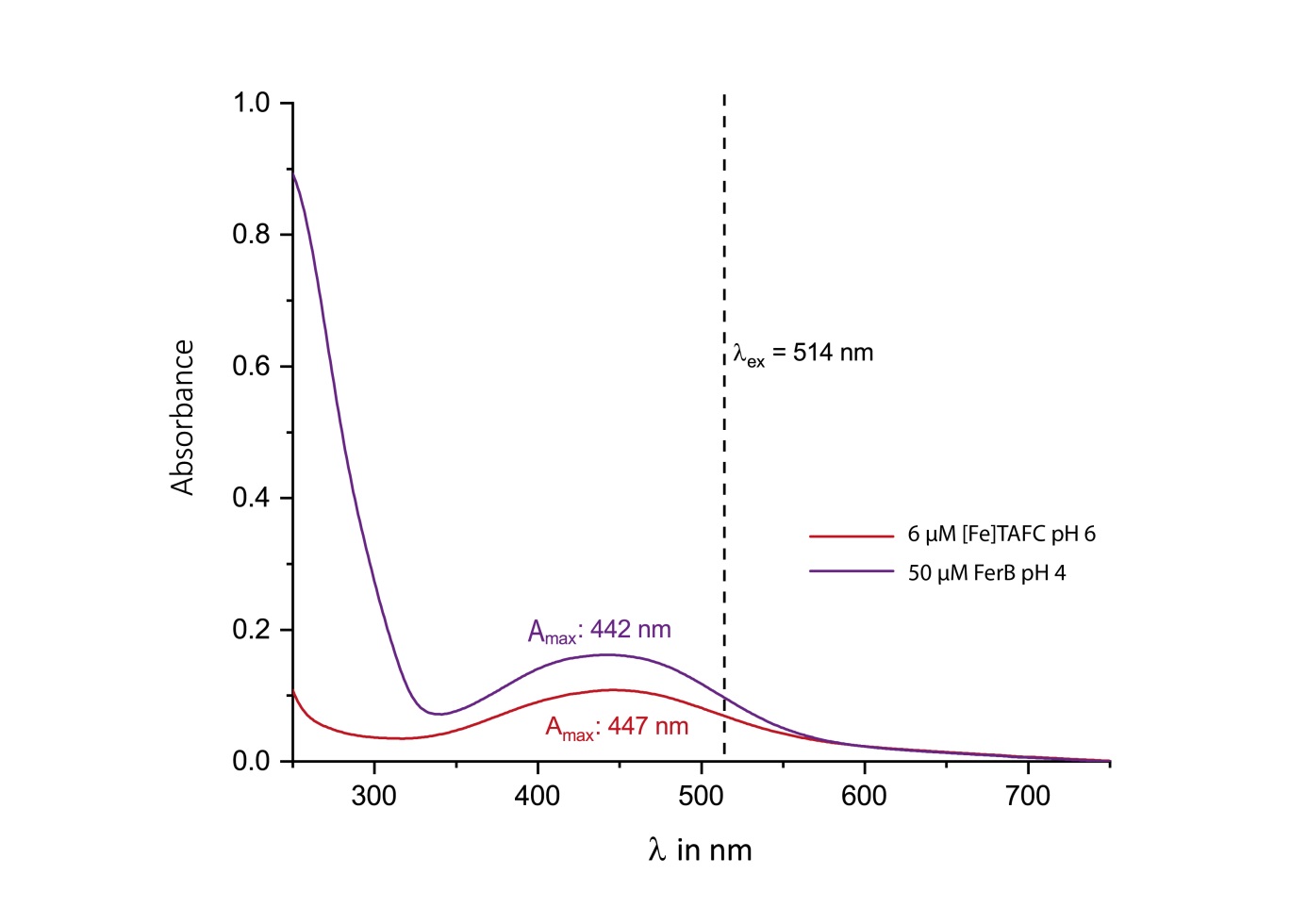


Fig. S1 UV-Vis spectra of [Fe]TAFC and ferrioxamine B measured in solution. The dashed line indicates the excitation wavelength used in the Raman spectroscopic study


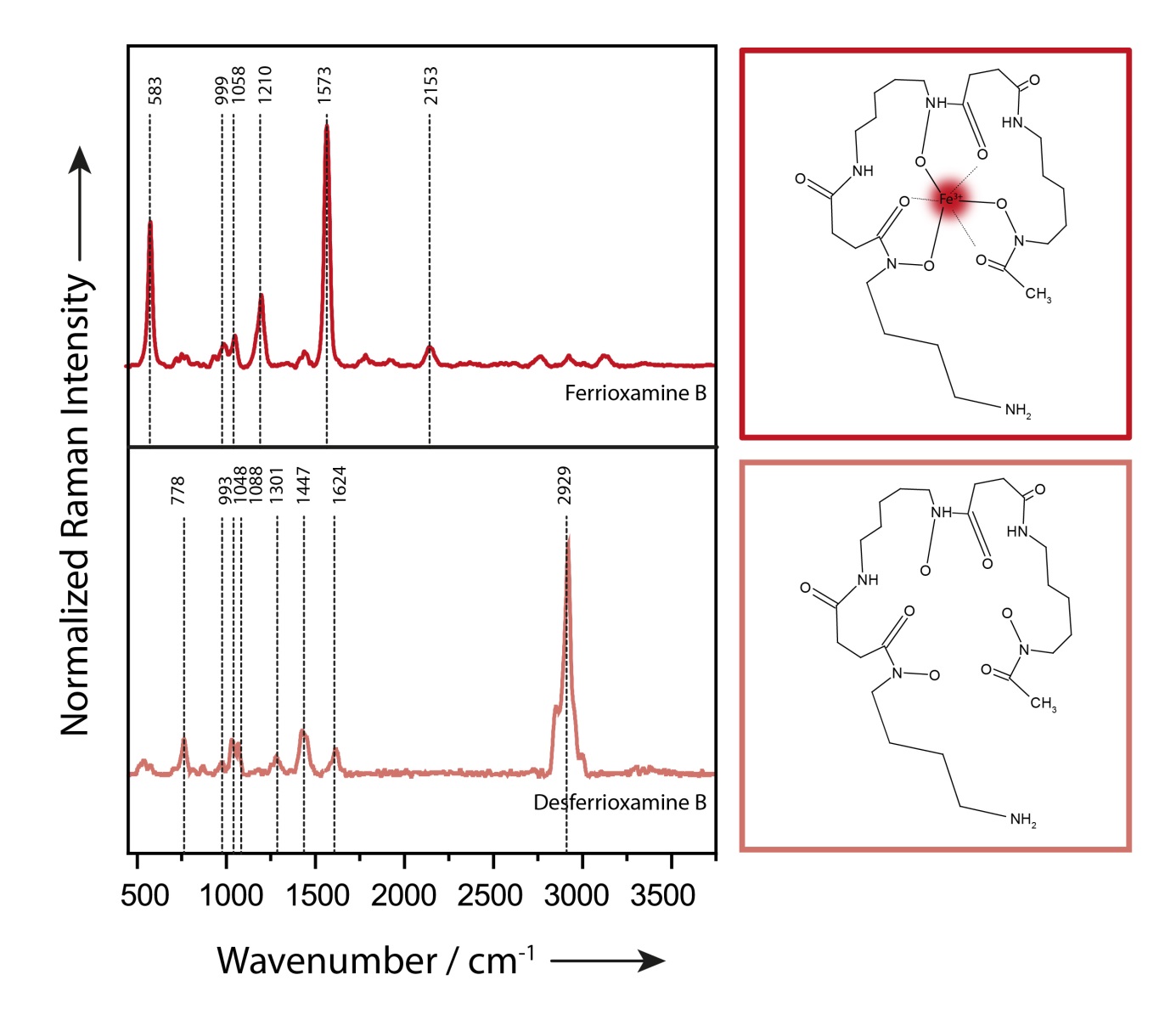


Fig. S2 Raman spectra and structural formulas of ferrioxamine B and desferrioxamine B. The band at 2153 cm^-1^ for FerB is most likely a combination band of the 583 cm^-1^ and 1573 cm^-1^ peaks


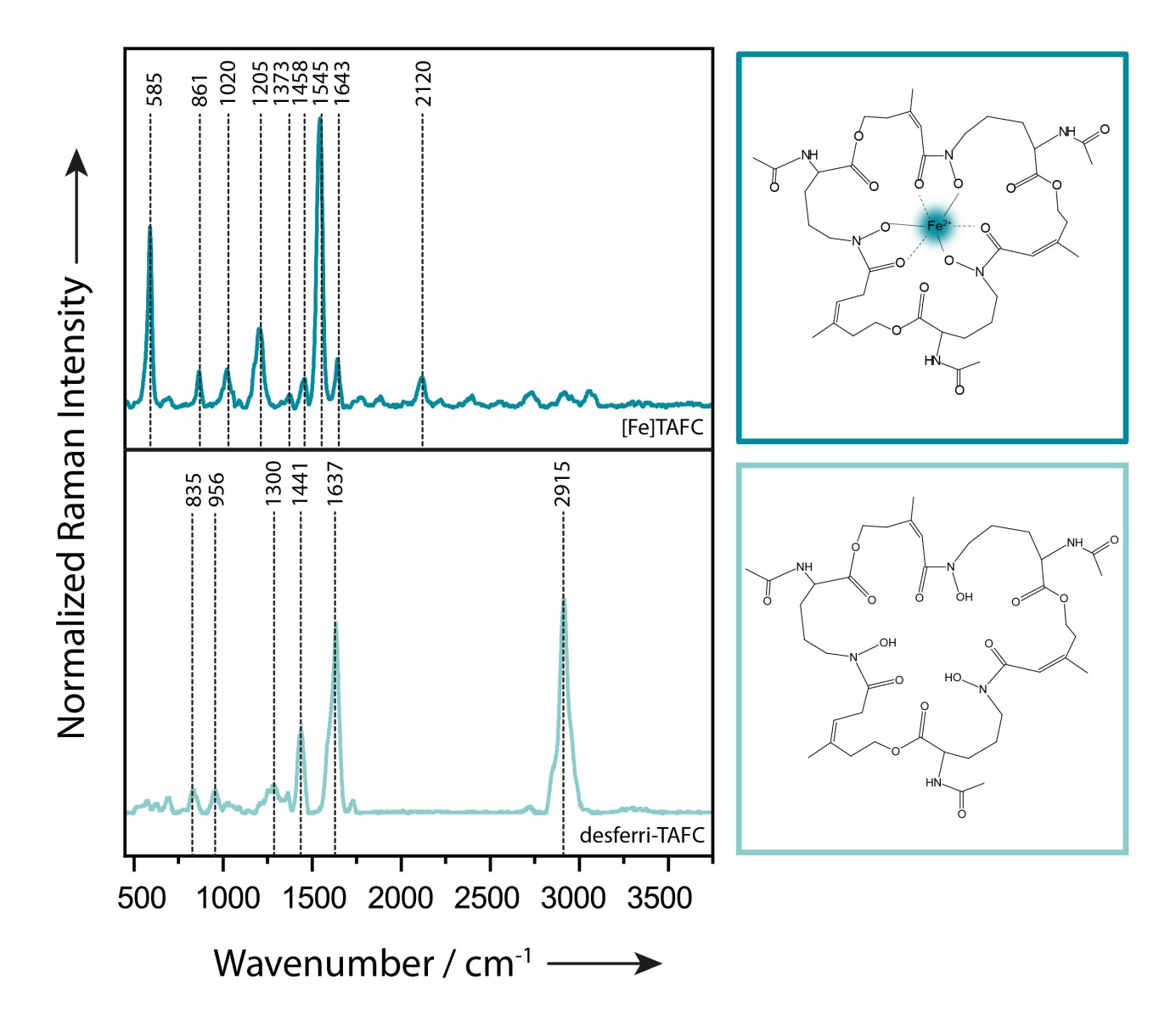


**Fig. S3** Raman spectra and structural formulas of [Fe]TAFC and desferri-TAFC. The band observed at 2120 cm^-1^ probably is a combination band resulting from the 1545 cm^-1^ and 585 cm^-1^ modes

Table S1 [Fe]TAFC masses, amounts of substance and concentrations for investigated samples

| **m([Fe]TAFC) in 0.75 µl** | **n([Fe]TAFC) in 0.75 µl** | **c([Fe]TAFC) in 0.75 µl** |
| --- | --- | --- |
| 100 ng | 110 pmol | 147 µM |
| 50 ng | 55.2 pmol | 73.6 µM |
| 25 ng | 27.6 pmol | 36.8 µM |
| 10 ng | 11.0 pmol | 14.7 µM |
| 5 ng | 5.52 pmol | 7.36 µM |
| 2.5 ng | 2.76 pmol | 3.68 µM |
| 1.5 ng | 1.66 pmol | 2.21 µM |
| 1 ng | 1.10 pmol | 1.47 µM |
| 0.5 ng | 0.55 pmol | 0.74 µM |
| 0 ng | 0 pmol | 0 µM |
| *M([Fe]TAFC) = 905.76 g/mol; M(TAFC) = 852.94 g/mol* | | |

**
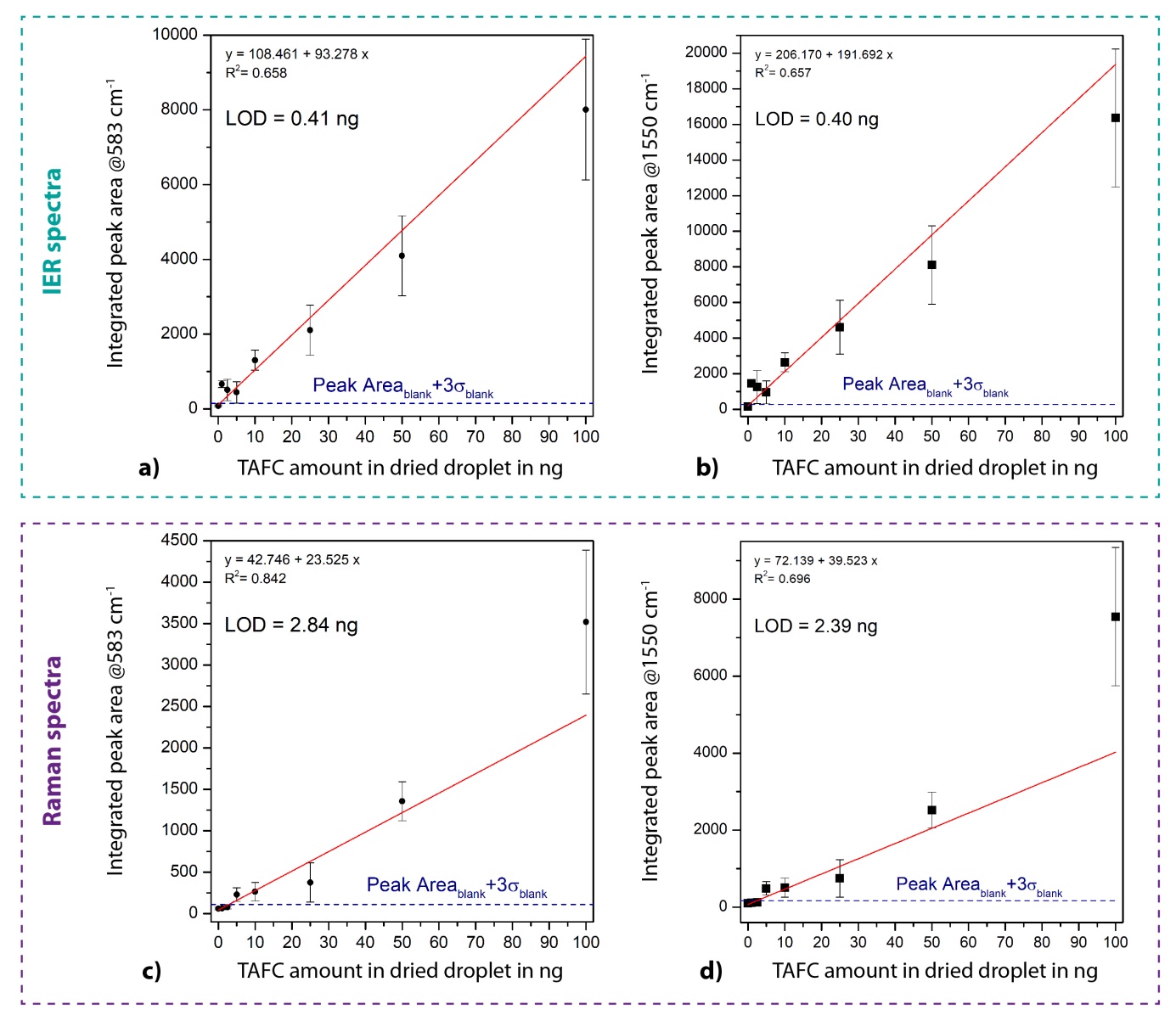
**

**Fig. S4** The peak area of the 583 cm^-1^ and 1550 cm^-1^ Raman mode as a function of [Fe]TAFC amount in the dried droplet with linear fitting. a) and b) IERS data set c) and d) Raman data set. The blue lines indicate the calculated LODs


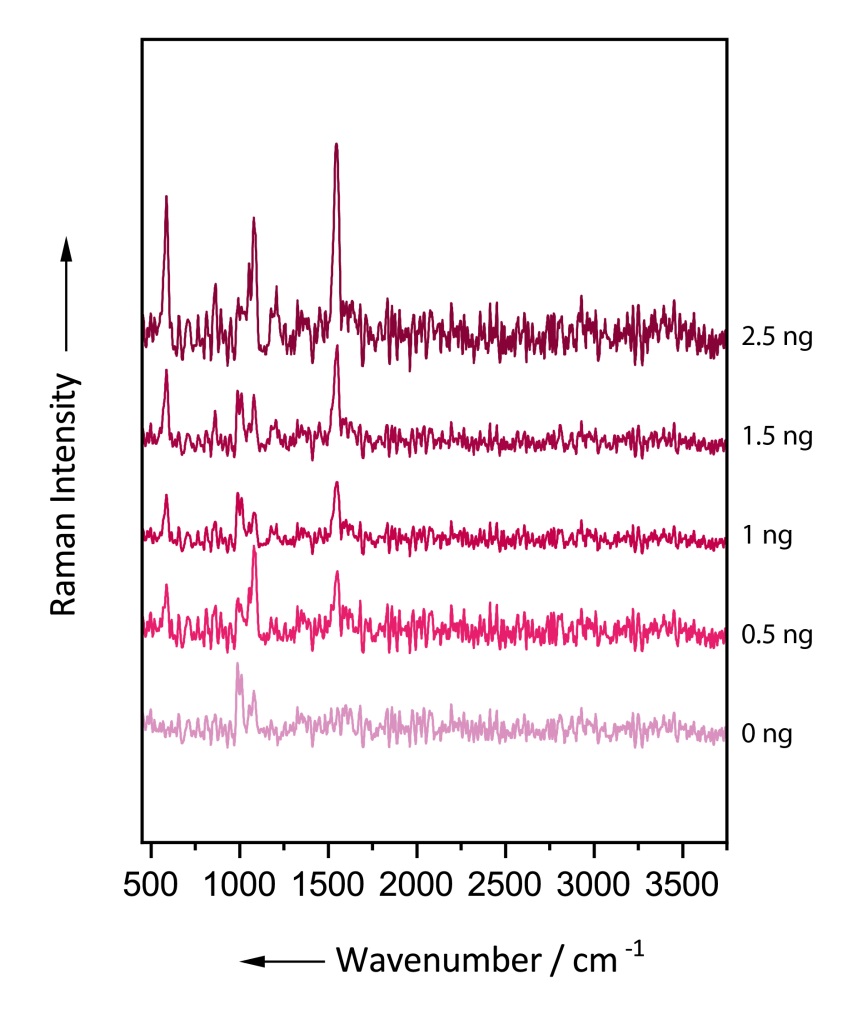


Fig. S5 Interference enhanced Raman (IER) mean spectra of drop dried of [Fe]TAFC samples. The amounts of [Fe]TAFC refer to a sample volume of 0.75 µl. The spectra have been shifted vertically for clarity


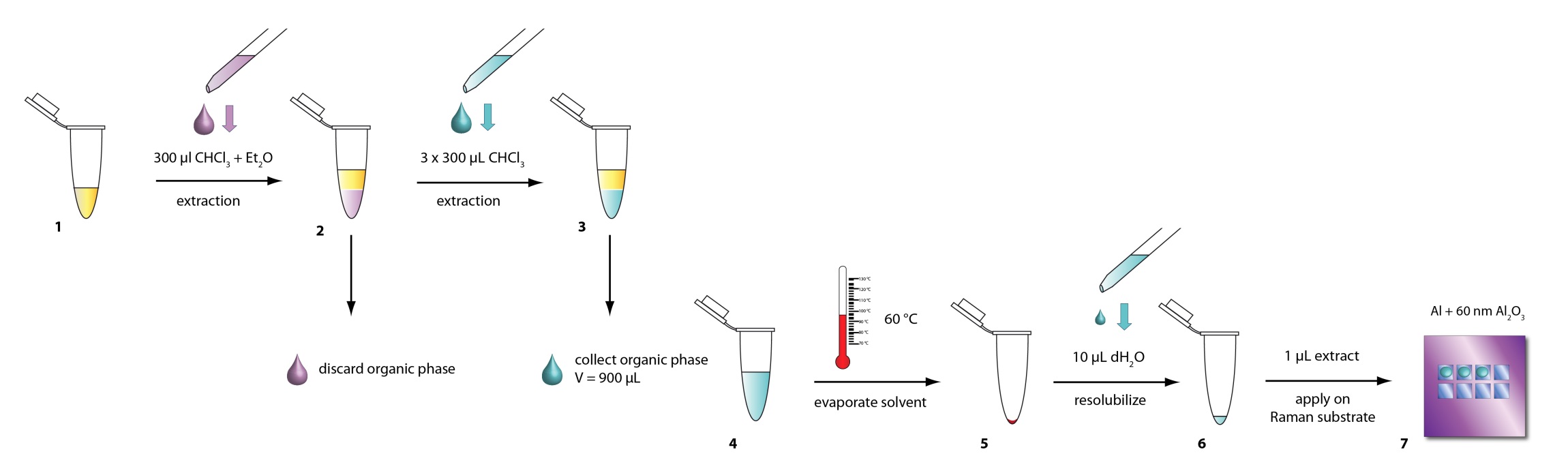


Fig. S6 Schematic display of the extraction protocol for [Fe]TAFC from urine samples


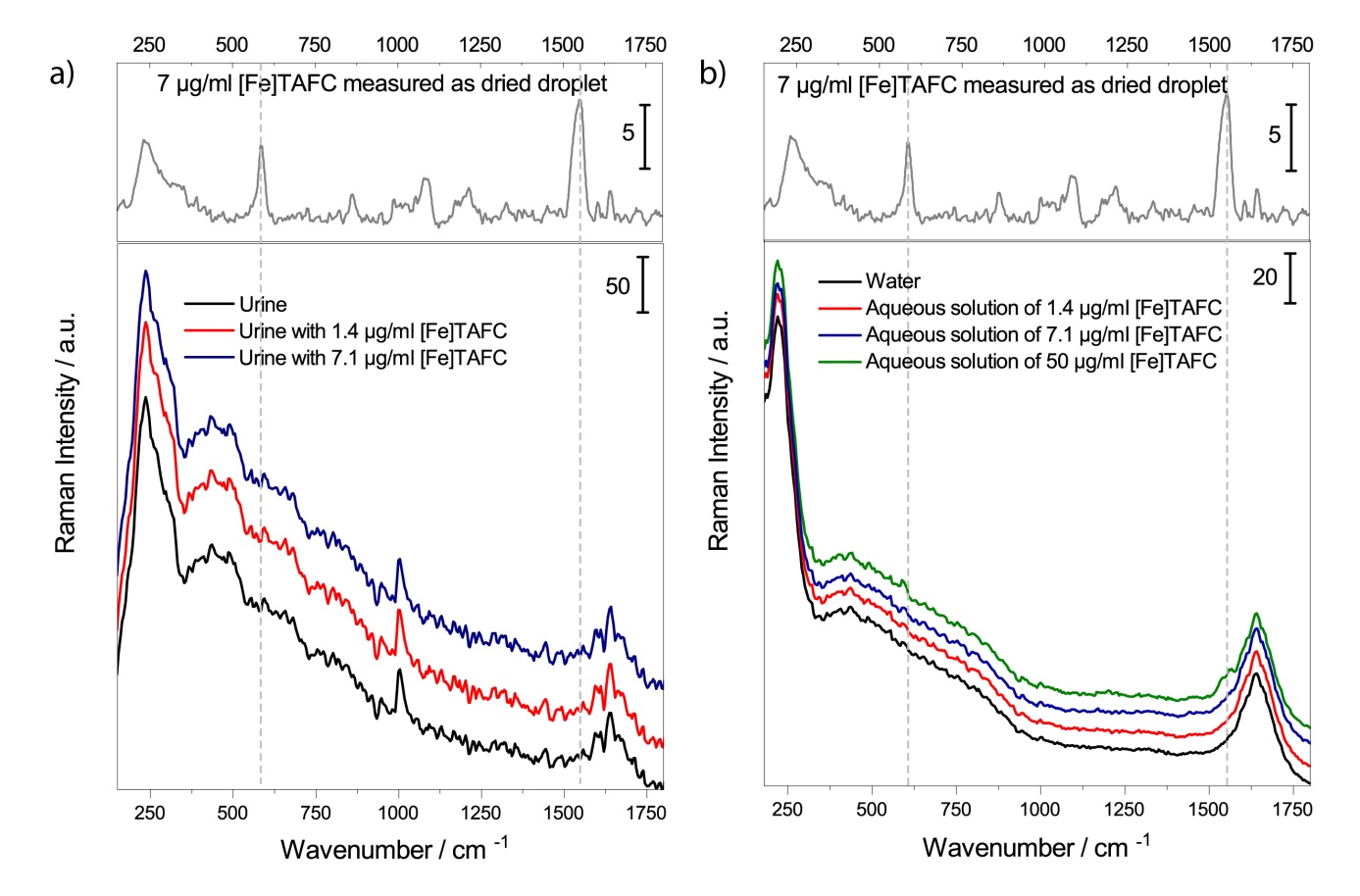


Fig. S7 Background corrected Raman mean spectra of [Fe]TAFC in solution: a) urine, b) purified water. Per sample 100 spectra in liquid phase were acquired using a 10x objective (NA = 0.25), 1s integration time and a laser power of 40 mW. For comparison, in the upper part of the diagrams a reference spectrum of a drop dried sample of [Fe] TAFC is included. The spectra in the lower part of the diagrams have been shifted for clarity

Table S2 [Fe]TAFC and ferrioxamine B masses, amounts of substance and concentrations for classification analysis

| **m([Fe]TAFC) in 0.75 µl** | **c([Fe]TAFC) in 0.75 µl** |
| --- | --- |
| 170 ng | 250 µM |
| 100 ng | 150 µM |
| 34 ng | 50 µM |
| 10 ng | 15 µM |
| **m(FerB) in 0.75 µl** | **c(FerB) in 0.75 µl** |
| 125 ng | 250 µM |
| 50 ng | 100 µM |
| 25 ng | 50 µM |
| 10 ng | 20 µM |
| *M([Fe]TAFC) = 905.76 g/mol; M(FerB) = 616.5 g/mol* | |


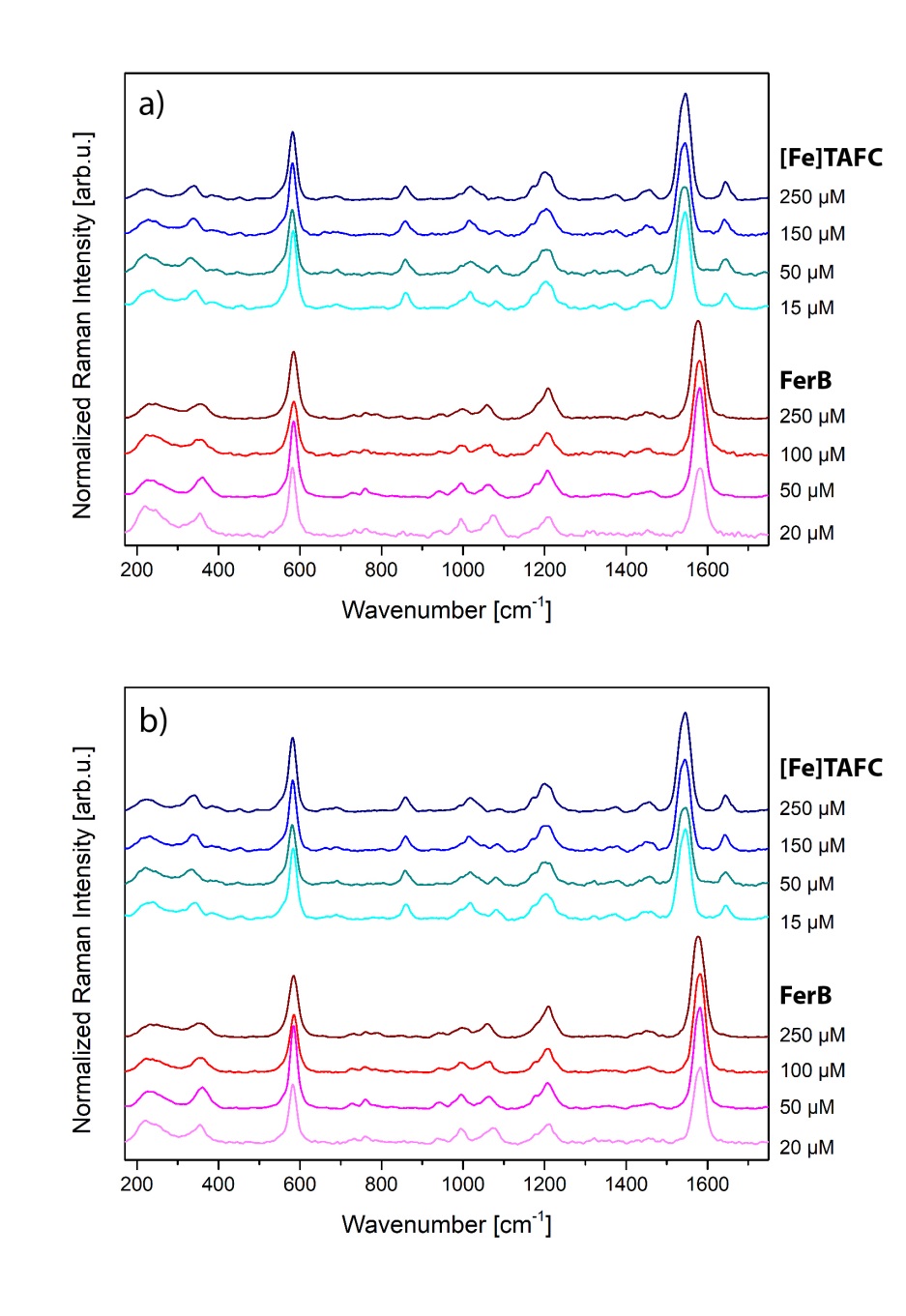


Fig. S8 Fingerprint regions of a) IER spectra and b) Raman spectra used for the differentiation of [Fe]TAFC and FerB. The spectra were shifted for clarity

Table S3 Classification results for a) IER spectra and b) Raman spectra

| **a)** | | | **TRUE** | | | | | | | |  | |
| --- | --- | --- | --- | --- | --- | --- | --- | --- | --- | --- | --- | --- |
|  |  |  | **FerB**  **[µM]** | | | | **[Fe]TAFC**  **[µM]** | | | |  |  |
|  |  |  | 100 | 20 | 250 | 50 | 150 | 15 | 250 | 50 | **Sens.** | **Spec.** |
| **PREDICTED** | **FerB**  **[µM]** | 100 | 295 | 1 | 0 | 0 | 0 | 0 | 0 | 0 | 98.33 | 99.95 |
|  |  | 20 | 0 | 299 | 0 | 0 | 0 | 0 | 0 | 0 | 99.67 | 100 |
|  |  | 250 | 0 | 0 | 300 | 1 | 0 | 0 | 0 | 0 | 100 | 99.95 |
|  |  | 50 | 5 | 0 | 0 | 299 | 0 | 0 | 0 | 0 | 99.67 | 99.76 |
|  | **[Fe]TAFC [µM]** | 150 | 0 | 0 | 0 | 0 | 288 | 0 | 0 | 0 | 96 | 100 |
|  |  | 15 | 0 | 0 | 0 | 0 | 11 | 300 | 0 | 0 | 100 | 99.48 |
|  |  | 250 | 0 | 0 | 0 | 0 | 1 | 0 | 300 | 0 | 100 | 99.95 |
|  |  | 50 | 0 | 0 | 0 | 0 | 0 | 0 | 0 | 300 | 100 | 100 |

| **b)** | | | **TRUE** | | | | | | | |  | |
| --- | --- | --- | --- | --- | --- | --- | --- | --- | --- | --- | --- | --- |
|  |  |  | **FerB**  **[µM]** | | | | **[Fe]TAFC**  **[µM]** | | | |  |  |
|  |  |  | 100 | 20 | 250 | 50 | 150 | 15 | 250 | 50 | **Sens.** | **Spec.** |
| **PREDICTED** | **FerB**  **[µM]** | 100 | 272 | 0 | 3 | 1 | 0 | 0 | 0 | 0 | 98.55 | 99.81 |
|  |  | 20 | 2 | 231 | 0 | 0 | 0 | 0 | 0 | 4 | 99.14 | 99.72 |
|  |  | 250 | 0 | 0 | 295 | 0 | 0 | 0 | 0 | 0 | 98.33 | 100 |
|  |  | 50 | 2 | 0 | 1 | 297 | 0 | 0 | 0 | 1 | 99 | 99.81 |
|  | **[Fe]TAFC [µM]** | 150 | 0 | 0 | 0 | 0 | 271 | 8 | 7 | 1 | 90.33 | 99.22 |
|  |  | 15 | 0 | 0 | 0 | 1 | 5 | 341 | 0 | 0 | 97.43 | 99.7 |
|  |  | 250 | 0 | 0 | 0 | 0 | 21 | 1 | 293 | 2 | 97.67 | 98.83 |
|  |  | 50 | 0 | 2 | 1 | 1 | 3 | 0 | 0 | 292 | 97.33 | 99.66 |

**
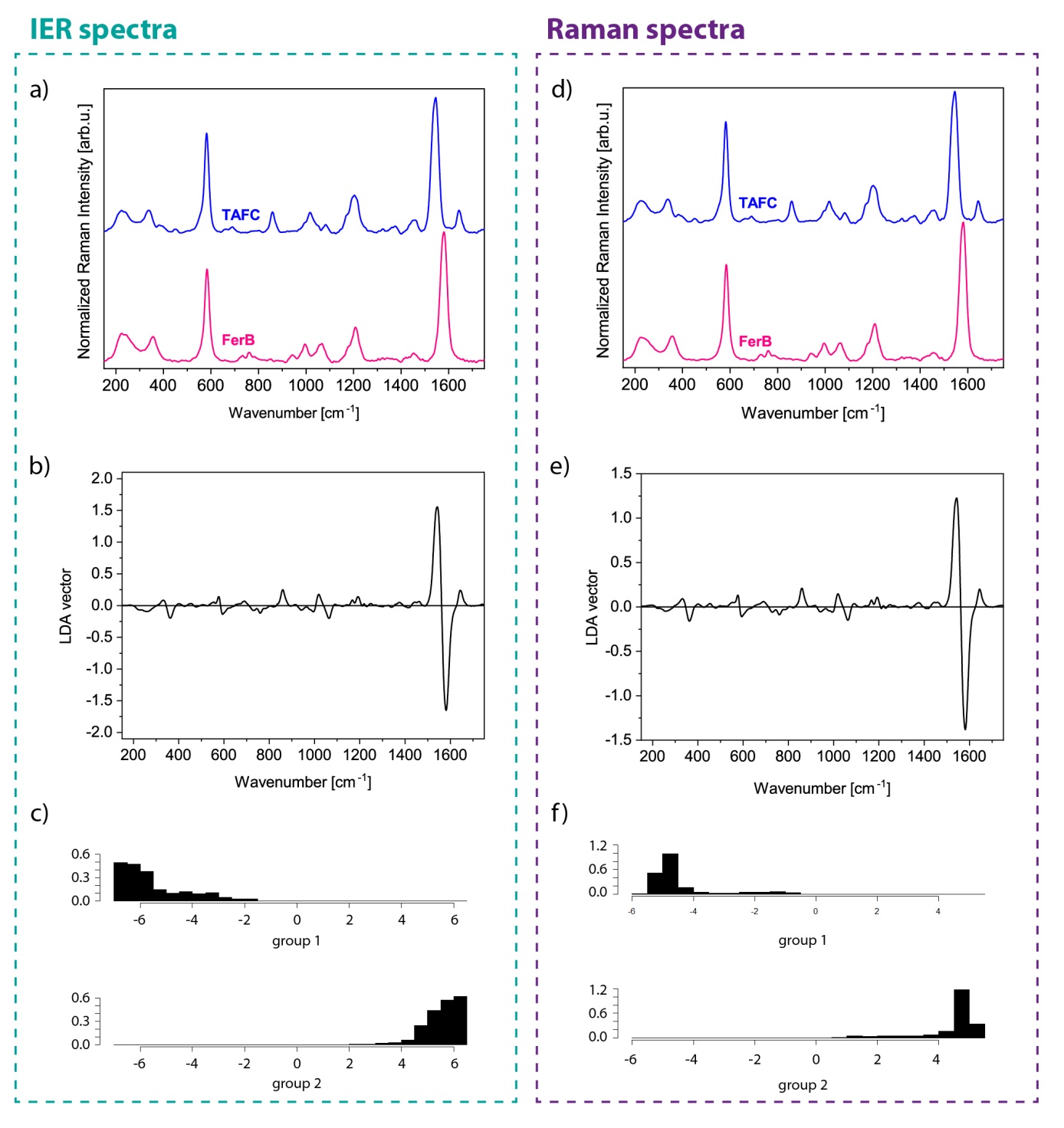
**

**Fig. S9** Mean spectra of [Fe]TAFC and FerB, LDA vector for two-class-classification with leave-one-concentration-out-cross-validation and corresponding LDA plot for IER spectra (a, b, c) and Raman spectra (d, e, f). An accuracy of 100% was achieved for both detection techniques
